# Clinical correlation of serum zinc and chromium levels in patients with type 2 diabetes mellitus and complications in Pakistan: a retrospective study

**DOI:** 10.7717/peerj.20184

**Published:** 2026-01-28

**Authors:** Humma Nayyar, Attya Bhatti, Peter John, Gohar Khan

**Affiliations:** 1 Department of Biomedicine/Atta Ur Rehman School of Applied Biosciences, National University of Science and Technology, Islamabad, Punjab, Pakistan; 2 NUST School of Health Sciences, National University of Science and Technology, Islamabad, Punjab, Pakistan

**Keywords:** Trace elements, Zinc and chromium, Diabetic complication, Diagnosis of trace elements, Glycemic control

## Abstract

**Background:**

Type 2 diabetes mellitus (T2DM) is a chronic metabolic disorder characterized by insulin resistance and hyperglycemia. The role of trace elements such as zinc and chromium in the pathophysiology of T2DM has garnered significant attention due to their involvement in glucose metabolism and insulin regulation.

**Objective:**

This cross-sectional study evaluated the clinical correlation between serum zinc and chromium levels in T2DM patients with and without complications in Pakistan.

**Methods:**

A total of 145 participants were included, comprising 100 T2DM patients (80 with complications: retinopathy, nephropathy, cardiovascular disease, and neuropathy; 20 without complications) and 45 healthy controls. Serum zinc and chromium levels were measured using atomic absorption spectrometry and their associations with glycated hemoglobin (HbA1c), demographic factors, and clinical profiles were evaluated.

**Results:**

The results showed that serum zinc and chromium levels were significantly lower in T2DM patients as compared to healthy controls (*p* value = 0.02 and *p* value = 0.001, respectively). Among diabetic subgroups, patients with diabetic neuropathy had the lowest zinc levels (*p* = 0.0001), while those with cardiovascular disease had significantly reduced chromium levels (*p* = 0.0002). Multivariate regression analysis showed that HbA1c levels were significantly associated with both zinc (β = 1.588, *p* value = 0.02) and chromium (β = 1.485, *p* value = 0.001), suggesting that deficiencies in these trace elements may contribute to poor glycemic control and the progression of diabetic complications.

**Conclusion:**

These findings highlight the potential role of zinc and chromium supplementation as an adjunctive therapeutic approach in managing T2DM and preventing its complications. Further longitudinal studies are needed to establish causality and explore the underlying mechanisms of trace element deficiency in diabetic patients.

## Introduction

Type 2 diabetes mellitus (T2DM) is a significant and growing health concern in Pakistan, often leading to various complications that adversely affect patient morbidity and mortality (Alotaibi et al., 2017; Aslam et al., 2022; Ma, 2018; Shi et al., 2018). Emerging evidences reveals that trace elements, particularly zinc and chromium, play crucial roles in the pathogenesis and progression of T2DM and its associated complications. These micronutrients are essential for insulin metabolism and glucose homeostasis, indicating that their deficiencies may exacerbate glycemic dysregulation in diabetic patients (Ahmad et al., 2024; Asghari et al., 2022; Moradi et al., 2019). Zinc is essential for insulin synthesis, storage, and secretion (Kloubert & Rink, 2015; MacKenzie & Bergdahl, 2022). Numerous studies have reported significantly lower serum zinc levels in T2DM patients compared to healthy individuals (Kant et al., 2021; Singh, Chandey & Mohan, 2025). For instance, Wang et al. (2024) demonstrated that reduced serum zinc levels were inversely correlated with glycated hemoglobin (HbA1c) levels, indicating poorer glycemic control. Similarly, Farooq et al. (2020) observed a negative association between zinc deficiency and HbA1c levels (reflects the percentage of red blood cells that have glucose bound to hemoglobin), highlighting the role of zinc in disease management.

Chromium plays a pivotal role in enhancing insulin activity and modulating carbohydrate, lipid, and protein metabolism (Mattos Pereira & Nair, 2024; Zhang et al., 2022). Serum chromium levels have consistently been found to be lower in patients with diabetes compared to non-diabetic individuals (Polat et al., 2024; Rajendran et al., 2015). A recent study reported that chromium supplementation in diabetic patients with low serum chromium levels improved glucose metabolism, suggesting a potential therapeutic role (Alkhalidi, 2023). Deficiency in chromium level can lead toward serious complications. Chromium deficiency in T2DM can lead toward cardiovascular diseases. Farrokhian et al. (2020) reported that chromium supplementation in T2DM patients with cardiovascular diseases improved oxidative stress, lipid profiles, glucose control, inflammatory markers, and weight regulation, underscoring its potential role in mitigating both metabolic disturbances and diabetic complications.

Despite these findings, there is a notable gap in literature addressing the clinical relevance of serum zinc and chromium levels in relation to T2DM complications within the Pakistani population. Given the unique dietary habits, genetic predispositions, and socioeconomic factors in Pakistan, it is essential to investigate how deficiencies in trace elements may influence the progression of T2DM and its complications. In South Asians, including those in Pakistan, insulin resistance often occurs at lower body mass indices, and micronutrient deficiencies may be more pronounced due to dietary imbalances and limited access to healthcare. Additionally, environmental exposures and cultural dietary preferences may result in trace element profiles that differ significantly from those reported in Western or East Asian populations. This retrospective study aims to assess the serum levels of zinc and chromium in T2DM patients with complications in Pakistan, exploring potential correlations between these micronutrient deficiencies and the severity or presence of diabetic complications. By examining trace element status in relation to diabetic complications, this research highlights the potential of zinc and chromium as biomarkers for risk stratification and as targets for nutritional interventions. These findings may support the development of targeted, context-specific strategies to enhance diabetes management and lessen the burden of complications in Pakistan.

## Materials & Methods

### Participants/study subjects

Patients were recruited from Federal Polyclinic Hospital (Islamabad, Pakistan) for a pilot study conducted between January–July 2024. Individuals diagnosed with T2DM were identified from the hospital’s diabetic outpatient registry. The proportion of diabetic patients attending the polyclinic during the study period was approximately 52%, ensuring a representative sample of the T2DM population. The National University of Science and Technology (NUST) Islamabad granted ethical approval to carry out the study within its associated hospital and facilities (Ethical Application Ref: 12-2023-ASAB-01/01). The sample size of 145 was determined based on the availability of participants within the study timeframe. Patients aged 35–70 years with a physician-confirmed diagnosis of T2DM according to the American Diabetes Association (ADA) criteria were included. Only patients with a minimum diabetes duration of 5 years were enrolled to ensure the potential presence of complications (ElSayed et al., 2023). Patients with other types of diabetes, chronic inflammatory diseases, or those on zinc/chromium supplementation were excluded. The control group consisted of individuals without diabetes, recruited from general health screenings to ensure that they did not have underlying metabolic disturbances that could confound the results. Participants were selected based on the following inclusion criteria: Fasting blood glucose (FBG) < 5.6 mmol/L, HbA1c < 5.7%, confirming non-diabetic status, no history of hypertension, cardiovascular disease, renal impairment, or neurological conditions, no current use of medications or supplements affecting glucose metabolism or trace element levels (*e.g.*, zinc or chromium supplements). Normal renal and liver function tests, based on available medical records or recent laboratory screening, no acute or chronic infections at the time of recruitment. Although smaller than initially planned, this sample size was sufficient to provide meaningful insights into the relationship between zinc and chromium levels and T2DM complications. Written informed consent form were taken from both patients and controls after full description of procedures and study objectives.

### Inclusion and exclusion criteria

Eligible participants were men and women between 35 and 70 years of age with a confirmed diagnosis of T2DM of more than 5 years’ duration. Both individuals without complications and those with a single diabetes-related complication (retinopathy, neuropathy, nephropathy without kidney failure, or cardiovascular disease) were considered. Diagnosis was established using standard biochemical criteria, including random blood sugar (RBS) > 11 mmol/L, fasting blood sugar (FBS) > seven mmol/L, and HbA1c > 7.1%. Patients were excluded if they had gestational diabetes, advanced kidney failure, nephropathy accompanied by kidney failure, more than one diabetes-related complication, or any other chronic or infectious condition apart from T2DM. Those with active infections at the time of recruitment or who were taking zinc or chromium supplements were also excluded. Inclusion and exclusion criteria for sample collection are described in Table 1

**Table 1:** Inclusion and exclusion criteria for participant selection. This table outlines the eligibility parameters applied during participant recruitment. Individuals were included if they met all predefined inclusion criteria and excluded if they met any of the specified exclusion criteria. These criteria ensured a well-defined and homogenous study population appropriate for valid statistical analysis.

Inclusion criteria	Exclusion criteria
Patients diagnosed with type 2 diabetes mellitus (T2DM).	Patients with gestational diabetes.
Duration of disease > 5 years.	Patients with end-stage kidney failure.
Age between 35–70 years.	Patients with infectious diseases or any chronic illness other than T2DM.
Random blood sugar (RBS) level > 11 mmol/L. Fasting blood sugar (FBS) level > 7 mmol/L. HbA1c > 7.1%.	Patients with active infections at the time of recruitment, to minimize confounding due to inflammation or altered metabolic parameters. Patients with multiple T2DM-associated complications (*i.e.*, more than one complication).
Presence of one of the following complications: Diabetic retinopathy, diabetic neuropathy, diabetic nephropathy (without kidney failure) and diabetic cardiovascular disease (CVD)	Patients with diabetic nephropathy accompanied by kidney failure. Patients currently taking zinc or chromium supplements (MacKenzie & Bergdahl, 2022).

### Diagnostic criteria and grouping according to complications

The subdivision of diabetic complications was based on the prevalence of each condition among the recruited participants, ensuring statistical feasibility for subgroup analyses. To minimize bias, patients with multiple complications were categorized according to their primary clinical diagnosis as determined by their treating physician. Sensitivity analyses were performed to assess the impact of overlapping conditions. Patients were recruited during routine diabetes management, complication screening, or follow-up visits. To reduce potential confounding, individuals with active infections at the time of recruitment were excluded, as infections could influence metabolic parameters and inflammatory markers.

Diabetic complications were diagnosed by using standard clinical and diagnostic criteria: **Diabetic retinopathy**: was diagnosed by funduscopic examination by an ophthalmologist using retinal imaging. **Diabetic cardiovascular disease (CVD)**: was defined based on clinical history of ischemic heart disease, angina, or heart failure, and confirmed through electrocardiogram (ECG). **Diabetic nephropathy**: was diagnosed by a combination of eGFR <60 mL/min/1.73 m^2^ and albuminuria >300 mg/day, with symptoms further evaluated using a validated nephropathy screening questionnaire. Duration of diabetes for patients is (≥7 years) **Diabetic neuropathy**: Diagnosed using a validated neuropathy symptom questionnaire supported by clinical signs, with nerve conduction studies performed in selected cases where further confirmation was necessary (ElSayed et al., 2023). Patients with severe kidney impairment (eGFR < 30 mL/min/1.73 m^2^ or requiring dialysis) were excluded to avoid confounding due to altered trace element metabolism, systemic inflammation, and medication effects.

**Figure 1:**
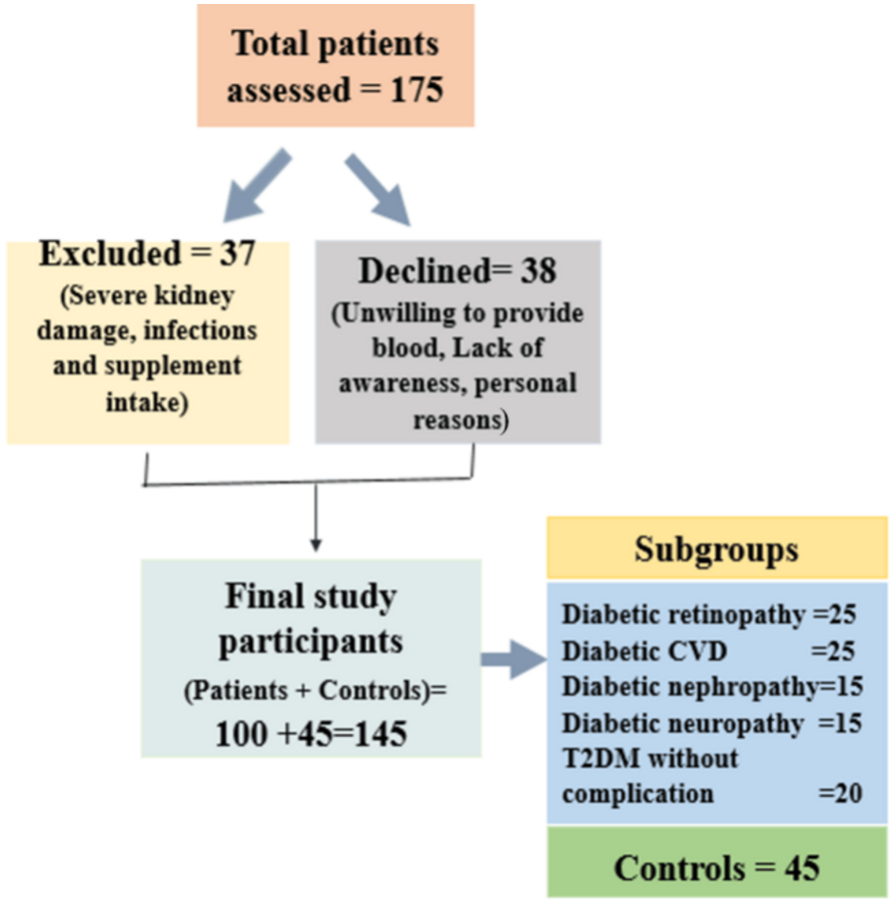
Participant enrollment and study group classification flowchart. The recruitment process and classification of study participants. A total of 175 patients were initially assessed. Of these, 37 individuals were excluded due to severe kidney damage, infections, or supplement intake, while 38 declined participation due to personal reasons, lack of awareness, or unwillingness to provide blood samples. The final sample included 145 participants, comprising 100 patients and 45 healthy controls. The patient group was further categorized into subgroups based on diabetic complications, including retinopathy (*n* = 25), cardiovascular disease (CVD) (*n* = 25), nephropathy (*n* = 15), neuropathy (*n* = 15), and those without complications (*n* = 20).

Based on preliminary pilot data, sample size calculation was performed using G*Power 3.1 software. Parameters were set at alpha level of 0.05, statistical power of 0.80, and a medium effect size (Cohen’s *d* = 0.5). These conditions yielded a minimum requirement of 90 participants to detect statistically significant differences in trace element levels. Although subgroup distribution was unequal, it reflected the natural prevalence of complications within the T2DM population. Despite these variations, each subgroup retained an adequate number of participants to permit meaningful statistical comparisons. However, the relatively smaller numbers in certain subgroups (neuropathy and nephropathy, *n* = 15 each) may have reduced the power for detecting subtle differences. Despite this limitation, the overall sample remained representative and statistically robust. Clinical and demographic data were analyzed to explore the relationship between serum zinc and chromium levels and the presence of T2DM-associated complications. The grouping of T2DM patients based on complications can be represented using a flowchart (Fig. 1)

### Biochemical parameters and clinical data

Biochemical parameters include glycated hemoglobin (HbA1c) which was measured using high performance liquid chromatography (HPLC), total cholesterol, triglycerides, high density lipoprotein (HDL), low density lipoprotein (LDL), urea and creatinine levels were measured using automatic biochemistry analyzer (Selectra Pro M biochemistry analyzer). Serum albumin levels were also measured by immune-assay techniques. Clinical data of T2DM patients were obtained through medical records and it comprised of sex, height age, body mass index (BMI), disease duration, history of smoking and hypertension. To address potential demographic or clinical mismatch between groups, a supplementary table has been included presenting baseline characteristics (such as age, BMI, gender, and blood pressure) across all subgroups, including the control group. This comparative analysis supports the validity of our findings by minimizing the likelihood that observed differences in trace element levels are confounded by key baseline variables (see Table S1).

### Serum sample preparation for trace elements measurement

Venous blood samples were collected after an overnight fast (8–12 h). All participants were instructed to avoid large meals and refrain from taking any supplements 24 h prior to sampling to minimize variation. Approximately five ml of blood samples was drawn by using sterile syringe under aseptic conditions following all the standard safety protocols. These blood samples was collected in sodium heparin tubes and subsequently centrifuged (Hermle Z 326 K Labortechnik GmbH, Berlin, Germany) at 4,000 rpm for 10 min to separate the serum (∼three mL).

Acid digestion is the most important step for trace element profiling through AAS (Atomic absorption spectrophotometer). To ensure long-term preservation of zinc and chromium in the form of stable complexes (*e.g.*, zinc chloride (ZnCl_2_) and chromium nitrate (Cr (NO_3_)_3_)), concentrated acids were added to the serum. Specifically, 1.5 mL of 65% nitric acid (HNO_3_; Cat. No. 695026; Sigma-Aldrich, St. Louis, MO, USA) was added to three mL of serum and the mixture was gently heated for 30–45 min. After cooling, 0.75 mL of 70% perchloric acid (HClO_4_; Cat. No. A229; Thermo Fisher Scientific, Waltham, MA, USA) was added, and heating was continued until dense white vapors were observed, indicating the completion of digestion. The mixture was then cooled, followed by the addition of six mL of deionized water. The sample was further heated until all vapors were expelled. Following digestion, the samples were transferred to Falcon tubes and stored at − 4 °C until trace element quantification by AAS to ensure sample stability.

### Measurement of trace elements

Serum zinc and chromium concentrations were determined using a Shimadzu AA-7000 Atomic Absorption Spectrophotometer (Shimadzu Corporation, Kyoto, Japan) equipped with hollow cathode lamps specific for each element. The digested samples were analyzed individually for Zn and Cr at wavelengths of 357.9 nm; lamp current 7 mA for zinc and 410 nm for chromium; lamp current 5mA. Readings were recorded *via* a computer interfaced with the spectrophotometer, and metal concentrations were calculated based on the corresponding standard calibration curves.

Calibration standards (Sigma-Aldrich, St. Louis, MO, USA) were prepared using serial dilutions in a matrix-matched solution to minimize interferences. Background correction techniques, such as deuterium lamp correction, were applied to eliminate non-specific scattering or absorption, following protocols described by Husakova et al. (2007). The atomization source was an air-acetylene flame, with an acetylene flow rate of 2.0 L/min and an air flow rate of 10.0 L/min. To account for potential matrix effects, the standard addition method was applied. Each sample was analyzed in triplicate, and concentrations were quantified using the instrument’s Shimadzu AA-7000 control software. Concentrations were computed using the instrument’s control software, which automated the measurement process and facilitated calibration curve integration. These procedures ensured accurate and reliable quantification of serum zinc and chromium levels using AAS.

### Standard solution preparation

The following steps were taken to get standard solutions for zinc and chromium:

### Stock solutions

Zinc and chromium stock solutions were prepared at a concentration of 1 g/L (1 mg/mL). The required amounts of pure zinc sulfate (ZnSO_4_⋅7H_2_O; Cat. No. Z4750; Sigma-Aldrich, St. Louis, MO, USA) and chromium nitrate (Cr(NO_3_)_3_⋅9H_2_O; Cat. No. 203874, Sigma-Aldrich, St. Louis, MO, USA) were dissolved in deionized water to achieve the desired concentrations.

### Intermediate standards

Intermediate standards were prepared by diluting the stock solutions to concentrations that reflect the expected levels in serum samples. For example, intermediate standards at concentrations of 10 mg/L and 100 mg/L were prepared.

### Working standards

Subsequently, working standards were prepared by further diluting the intermediate standards to concentrations within the expected physiological range (0.1 to 10 mg/L) for zinc and chromium in serum samples. All dilutions were performed using deionized water to ensure precision and accuracy in the measurements.

### Acid matrix matching

To ensure analytical accuracy and minimize matrix effects, the standard solutions were prepared with acid concentrations comparable to those of the digested serum samples. Since the serum samples were digested using nitric acid and perchloric acid, the standard solutions were also supplemented with the same volumes of 65% HNO_3_ and 70% HClO_4_ used during sample preparation. This approach ensured consistency between the sample and standard matrices, thereby improving the reliability of trace element quantification.

### Calibration curve

To generate the calibration curve, a series of standard solutions at varying concentrations were prepared, including at least five points covering the expected concentration range. These standards were then analyzed using the atomic absorption spectrophotometer to construct the calibration curve. Instrument settings were carefully optimized to ensure accurate detection of both chromium and zinc.

### Quality control procedures

The AAS was calibrated using certified reference standards for zinc and chromium prior to sample analysis. Quality control was maintained by analyzing standards at regular intervals to ensure accuracy and reproducibility of measurements .Blank controls (acid-digested deionized water) were included in every batch to detect potential contamination. Spiked recovery tests were performed by adding known amounts of zinc and chromium standards to selected serum samples, with recovery rates maintained within 95–105%. Instrument calibration was re-verified at regular intervals using mid-range standards. These measures ensured the reliability, precision, and reproducibility of zinc and chromium measurements in serum samples.

### Statistical analysis

G*Power 3.1 software for sample size calculation. SPSS 22.0 (IBM Corp., Armonk, NY, USA) was used to analyze the results. Graph pad prism were used to make graphs. The normality of data was assessed by using the Shapiro–Wilk test. Quantitative variables such as serum zinc and chromium levels were treated as continuous variables without transformation. Variables with normal distribution are presented as mean ± standard deviation (SD), while non-normally distributed data (*e.g.*, serum chromium) are presented as median and interquartile range (IQR). For comparison between more than two groups, one-way ANOVA was used for normally distributed variables, and the Kruskal–Wallis test was used for non-normal variables. Pairwise comparisons between two groups were performed using the independent *t*-test or Mann–Whitney U test, depending on data distribution.

A multivariate regression model was used to adjust for potential confounders such as age, gender, and BMI, ensuring that the observed relationships between trace element levels and complications. The inter element correlation (Zn and Cr) were analyzed using Pearson’s correlation for normally distributed data and Spearman’s correlation for non-normally distributed data. *P* < 0.05 was called as statistically significant.

## Results

### Demographic and biochemical parameters of the participants with T2DM and its associated complications

A total of 145 participants were included in the study, comprising 100 individuals with T2DM and 45 healthy controls. Of the total participants, 74 were male and 71 were female. Some individuals (not included in the study) declined to provide blood samples and data due to a lack of awareness, limited education, perceived absence of personal benefit, and competing health priorities. The mean age of T2DM participants was 58.0 ± 12.14 years. Among them, males had a mean age of 59.6 ± 12.83 years (50.5%), and females had a mean age of 56.91 ± 11.1 years (51.5%).Zinc and chromium level were determined for all participants. Demographic and clinical characteristics of T2DM patients and controls subjects were compared statistically (Table 2)

**Table 2:** Comparison of demographic and clinical characteristics between T2DM patients and healthy controls. The table presents a comparative analysis of clinical parameters between T2DM patients and control groups. Significant differences were observed in body mass index, serum total cholesterol, smoking history, hypertension, blood urea nitrogen, creatinine, and serum C- reactive protein. Additionally, zinc and chromium levels were significantly lower in T2DM patients (*P* < 0.05). (See Appendix S1).

Parameters	Control group (*n* = 45)	T2DM all data excluded grouping (*n* = 100)	*P value*
Males, n (%)	23 (53.4%)	51 (50.5%)	0.410
Females, n (%)	22 (46.5%)	49 (49.4%)	0.321
Age (years)	53.4 ± 9.14	58.02 ± 12.14	0.064
Hypertension, n (%)	5 (25%)	19 (45%)	<0.01
BMI	21.5 ± 4.59	22.5 ± 4.5	0.064
FBG (mmol /L)	5.0 (0.4–0.6)	8.3 (2.8–3.4)	<0.01
RBG (mmol/L)	6.7 (1.1–1.4)	11.8 (3.9–4.78)	<0.01
HbA1c (%)	5.2 ± 0.5	8.18 ± 1.65	0.001
Smoking, n (%)	3 (15%)	5 (25%)	0.03
Cholesterol (mmol/L)	4.6 (3.6–5.6)	6.6 (5.4–7.9)	0.04
Triglycerides (mmol/L)	1.1 (0.6–1.7)	2.02 (1.7–2.6)	0.01
Serum zinc	1.875 ± 0.984	1.06 ± 0.627	0.012
Serum chromium	0.276 ± 0.15	0.07 ± 0.132	0.001
Serum creatinine (mg/L)	0.9 ± 0.2	1.2 ± 0.5	0.01
Serum albumin (g/dl)	4.3 ± 0.5	1.0 ± 0.4	0.41
Serum urea (mg/dl)	25 ± 5	45 ± 12	0.03
Serum ALT (U/L)	15 ± 5	23 ± 13.5	0.14
Serum ALP (U/L)	60 ± 20	85 ± 30	0.05
Serum C-reactive protein (mg/L)	2.5 ± 2.0	3.23 ± 2.5	0.04
HDL	1.6 (1.0–2.1)	2.89 (1.5–2.3)	0.01
LDL	2.6 (1.6–3.6)	2.3 (1.8–3.9)	0.32

Based on clinical and demographic data, T2DM patients were further categorized according to the presence of complications. Of the 100 T2DM patients, 20 had no complication, 25 had diabetic cardiovascular disease (CVD), 25 had diabetic retinopathy, 15 had diabetic neuropathy, and 15 had diabetic nephropathy. The control group (*n* = 45) was age- and sex-matched with T2DM participants. No statistically significant differences were observed in age (*p* = 0.064) or BMI (*p* = 0.064) between groups (Table 2), ensuring appropriate demographic comparability. Clinical differences reflect disease status rather than baseline mismatch. The clinical parameters of these subgroups were compared with the control group to identify significant differences. Patients with diabetic nephropathy showed marked elevations in urea, creatinine, and uric acid levels compared to other groups. Those with diabetic neuropathy exhibited higher HbA1c levels, lower HDL cholesterol, and a greater prevalence of hypertension. Patients with diabetic CVD presented elevated total cholesterol, serum C-reactive protein, and lipid profile abnormalities. In contrast, patients with diabetic retinopathy did not demonstrate statistically significant clinical differences when compared to other complication subgroups.

### Serum zinc and chromium level in T2DM patients and other associated complications

**Figure 2:**
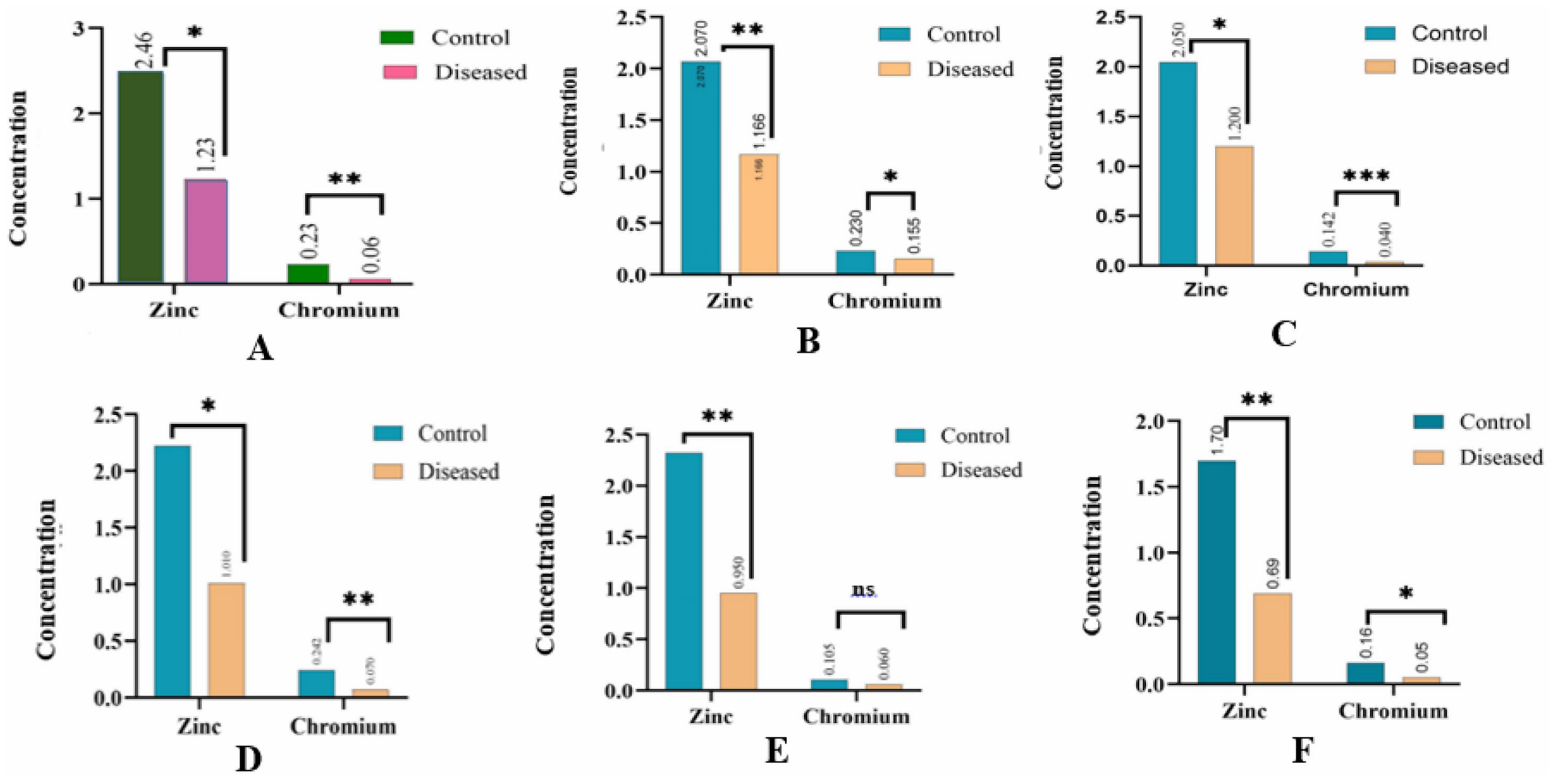
Serum concentrations of zinc and chromium in controls and T2DM subgroups. Comparison of serum zinc and chromium concentrations between control subjects and diabetic individuals, including subgroups with specific complications. (A) Overall comparison shows a significant reduction in both trace elements in diabetic patients *vs.* controls. (B) Diabetic retinopathy group exhibits significantly lower zinc and chromium levels, indicating a possible link to ocular complications. (C) Patients with cardiovascular disease also show marked deficiencies in both elements. (D) The diabetic nephropathy subgroup exhibited the most pronounced reductions in trace element levels, indicating a greater degree of imbalance. (E) In diabetic neuropathy, zinc is significantly reduced; chromium levels show no significant difference (ns). (F) Patients without complications display lower but less pronounced reductions zinc is significantly decreased, while chromium shows modest significance.Values are expressed as mean concentrations (*p < 0.05, **p < 0.01, ***p < 0.001, ns = not significant). The trend indicates consistent trace element deficiencies in diabetic patients, especially those with complications.

Out of 145 participants included in the study, 100 were patients with T2DM, and 45 were healthy controls. Among the total participants, 74 were male and 71 were female. Serum zinc and chromium levels (measured in mg/L) were determined for all participants and statistically compared between groups. The mean serum zinc level in the control group was 2.46 ± 0.954 mg/L, whereas in T2DM patients, it was significantly lower at 1.23 ± 0.597 mg/L (p 0.04), indicating a statistically significant difference. The median serum chromium level in T2DM patients was 0.06 mg/L (IQR: 0.03–0.12), compared to 0.234 mg/L (IQR: 0.18–0.29) in the control group (*p* < 0.01), also indicating a significant reduction. Figure 2A illustrates the reduced zinc and chromium levels in T2DM patients compared to controls.

Based on the subgrouping of 100 T2DM patients and 45 healthy controls, 20 T2DM patients without complications were compared with 10 age- and sex-matched healthy individuals. The mean serum zinc level in the control group was 2.07 ± 0.74 mg/L, while T2DM patients without complications had a significantly lower level of 1.166 ± 0.597 mg/L (*p* = 0.004), indicating a statistically significant difference. The median chromium level in the control group was 0.23 mg/L (IQR: 0.18–0.29), compared to 0.155 mg/L (IQR: 0.08–0.22) in the T2DM group (*p* = 0.03), also reflecting a statistically significant reduction. Figure 2B illustrates the serum levels of zinc and chromium in T2DM patients without complications compared to healthy controls. Similarly, 25 T2DM patients with cardiovascular disease (CVD) were compared with 11 healthy controls. The mean serum zinc level in controls was 2.05 ± 0.736 mg/L, while T2DM patients with CVD had a significantly lower level of 1.20 ± 0.787 mg/L (*p* = 0.012). The median chromium level in controls was 0.14 mg/L (IQR: 0.11–0.17), compared to 0.04 mg/L (IQR: 0.03–0.05) in T2DM patients with CVD (*p* = 0.0002), representing a highly significant reduction in chromium levels. As illustrated by Fig. 2C

Twenty-five T2DM patients with diabetic retinopathy were compared with 10 healthy controls. Serum zinc and chromium levels were compared between the two groups. The mean serum zinc level in the control group was 2.219 ± 0.51 mg/L, while T2DM patients with retinopathy exhibited a significantly lower level of 1.01 ± 0.54 mg/L (*p* = 0.02), indicating a statistically significant difference. The median serum chromium level in the control group was 0.242 mg/L (IQR: 0.18–0.31), while in T2DM patients with retinopathy it was markedly reduced to 0.07 mg/L (IQR: 0.05–0.11). This difference was found to be highly statistically significant (*p* = 0.001). As shown in Fig. 2D, the graph presents the serum levels of zinc and chromium in T2DM patients with retinopathy compared to healthy controls. Fifteen T2DM patients with diabetic nephropathy were compared with seven healthy controls. Serum zinc and chromium levels were assessed between the groups. The mean serum zinc level in the control group was 2.32 ± 0.68 mg/L, whereas T2DM patients with nephropathy showed a significantly lower level of 0.95 ± 0.56 mg/L (*p* = 0.01), indicating a statistically significant difference. The median serum chromium level in the control group was 0.105 mg/L (IQR: 0.07–0.19), while in T2DM patients with nephropathy it was 0.06 mg/L (IQR: 0.04–0.09). The difference in chromium levels between the two groups was not statistically significant (*p* = 0.47). As shown in Fig. 2E, the graph presents the serum levels of zinc and chromium in T2DM patients with nephropathy compared to healthy controls

Fifteen T2DM patients with diabetic neuropathy were compared with seven healthy controls. Serum zinc and chromium levels were analyzed between the groups. The mean serum zinc level in the control group was 1.70 ± 0.48 mg/L, while patients with neuropathy had a significantly lower level of 0.69 ± 0.381 mg/L (*p* = 0.006), indicating a highly significant difference. The median serum chromium level in the control group was 0.16 mg/L (IQR: 0.13–0.20), whereas in T2DM patients with neuropathy, it was 0.05 mg/L (IQR: 0.03–0.08). This reduction was statistically highly significant (*p* = 0.003). As depicts in Fig. 2F, the graph presents the serum levels of zinc and chromium in T2DM patients with neuropathy compared to healthy controls

### Comparison among all T2DM complications

**Figure 3:**
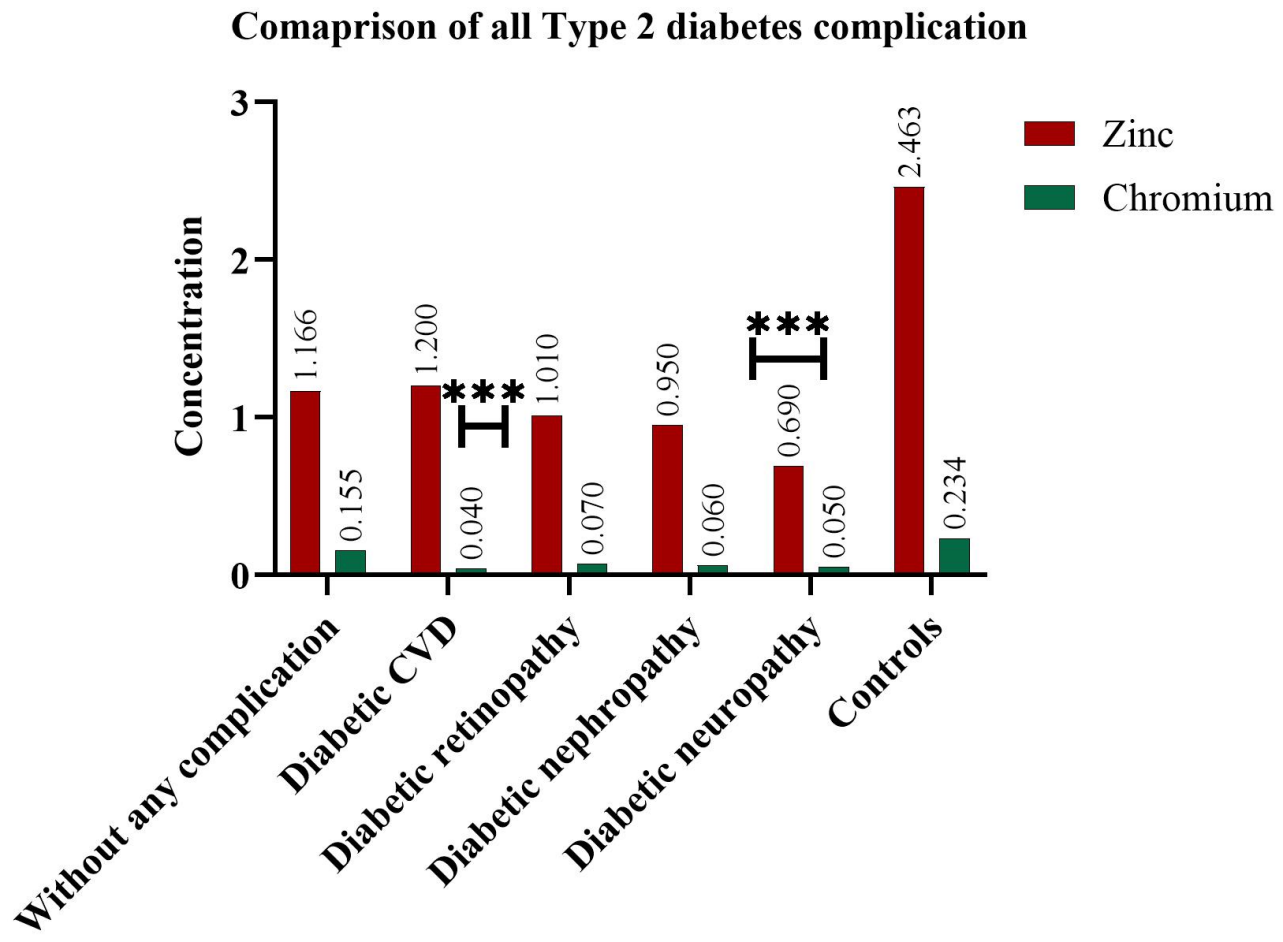
Comparative analysis of serum zinc and chromium levels across T2DM subgroups. The mean serum concentrations of zinc and chromium across different subgroups of T2DM, including patients with no complications, diabetic cardiovascular disease (CVD), retinopathy, nephropathy, neuropathy, and healthy controls. All diabetic subgroups exhibit reduced levels of both elements, with the diabetic neuropathy and retinopathy subgroups showing the most pronounced deficiencies, particularly in chromium, as denoted by *** (*p* < 0.001). These findings suggest a consistent trend of trace element depletion associated with the presence and severity of diabetic complications.

Serum zinc and chromium levels were compared across all T2DM complication subgroups. Among these, the lowest serum zinc levels were observed in patients with diabetic neuropathy, showing a highly significant difference (p = 0.0004) compared to controls. In contrast, serum chromium levels were most significantly reduced in patients with diabetic cardiovascular disease (CVD) (*p* = 0.0001). These findings suggest that zinc deficiency may be particularly associated with the development of diabetic neuropathy, while chromium deficiency may contribute to the progression of cardiovascular complications in T2DM patients. A comparison between the different groups of T2DM complications is illustrated in Fig. 3

### Inter-element co-relation between T2DM and controls

**Figure 4:**
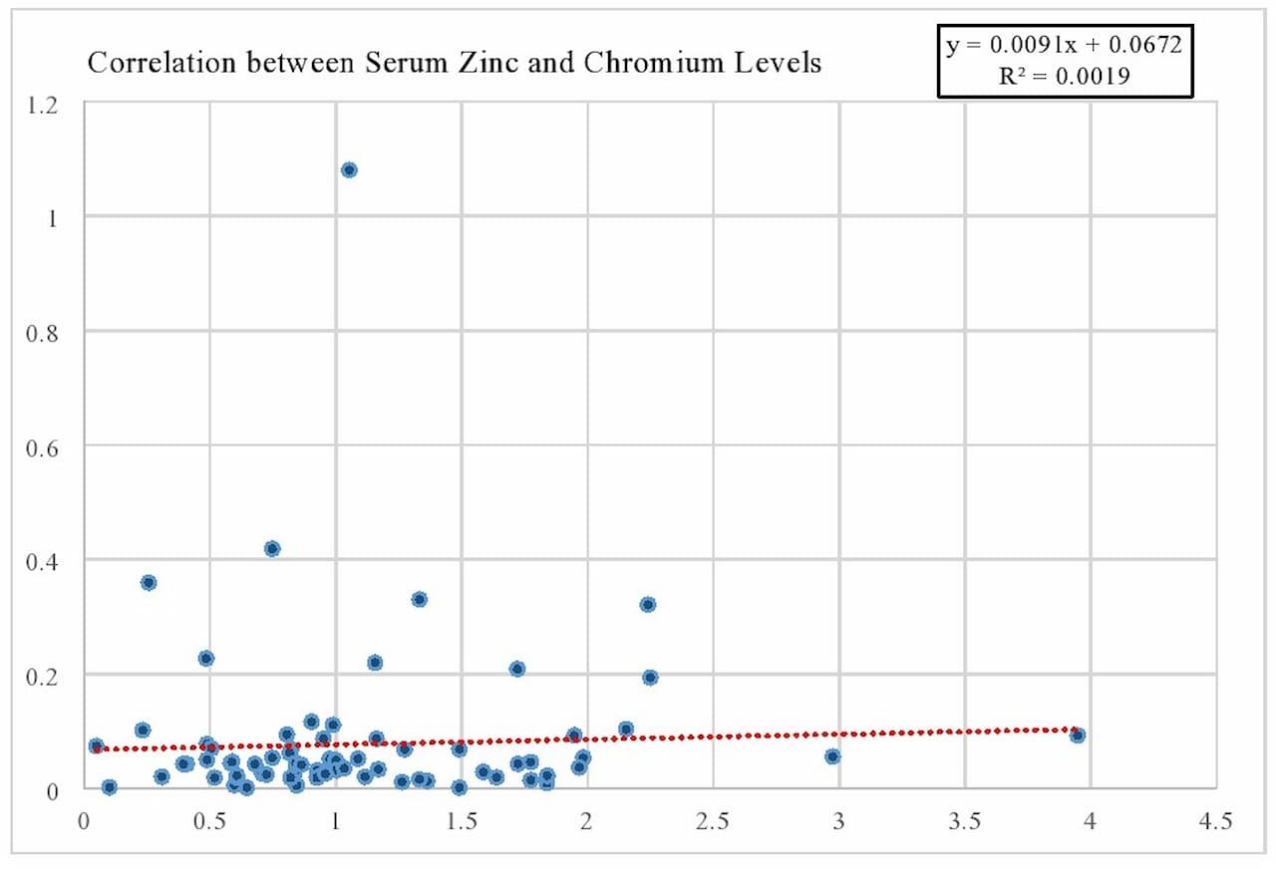
Scatter plot showing the correlation between serum zinc and chromium levels. Each dot represents an individual sample. A linear trend-line (dotted red line) is fitted to the data, with the equation *y* = 0.0091x+0.0672y = 0.0091x + 0.0672 *y* = 0.0091x + 0.0672 and coefficient of determination *R*^2^ = 0.0019 indicating a minimal linear association between the two trace elements.

The correlation between serum zinc and chromium levels was compared in both T2DM patients and healthy controls as illustrated by Table 3. A weak but statistically significant positive correlation was observed in the control group (*r* = 0.14121, *p* = 0.01). In contrast, the diseased group exhibited a statistically significant yet negligible negative correlation (*r* =  − 0.00146, *p* = 0.00023).These statistical findings are visually represented in the scatter plot, which displays individual data points along with a linear trend line, as depicted in Fig. 4 The plot reveals a minimal overall association between the two trace elements, with an *R*^2^ value of 0.0019, further confirming the weak correlation. This suggests that zinc and chromium levels do not strongly co-vary, particularly in the diabetic group, and may play independent roles in the progression of T2DM.

**Table 3:** Correlation between serum zinc and chromium level. Inter-element correlation between the T2DM patients and controls depicts the positive correlation with highly significant values.

Trace elements	Controls		Diseased	
	**r**	**P**	**R**	**P**
Zinc and chromium	0.14121	0.01	−0.00146	0.00023

### Correlation between Zn, Cr concentration and biochemical charactertics of T2DM complications

According to serum zinc levels were significantly and positively associated with disease duration, HbA1c, total cholesterol, fasting blood glucose (FBG), and random blood glucose (RBG). Similarly, serum chromium levels showed significant positive associations with HbA1c, total cholesterol, FBG, RBG, and serum creatinine. It can be illustrated by Table 4 In addition, a positive association was observed between zinc and chromium levels, suggesting a potential interplay in their regulation under diabetic conditions. These metabolic parameters are well-established indicators of poor glycemic control and are closely linked to the risk and severity of diabetic complications, including cardiovascular disease (CVD), retinopathy, nephropathy, and neuropathy.

**Table 4:** Multivariate correlation analysis between serum zinc and chromium level in diabetic complications. Zinc and chromium level were correlated with different demographical and clinical parameters. BMI, Body mass index; HbA1c, Glycated hemoglobin; LDL, Low density lipoprotein, BUN; Blood urea nitrogen, UA; Uric acid, RBG; Random blood glucose; FBG, Fasting blood glucose, HDL; High density lipoprotein.

T2DM patients with associated complications (CVD, retinopathy, neuropathy and nephropathy)						
	*Multivariate Regression analysis*					
Parameters	***Serum zinc***			***Serum chromium***		
	**β**	***95% Cl***	***P*** ** value**	**β**	***95% Cl***	***P*** **value**
Age (Years)	0.160	(−0.05, 0.37)	0.12	0.379	(−0.12, 0.88)	0.15
BMI (kg/m^2^ )	0.093	(−0.02, 0.21)	0.09	0.084	(−0.01, 0.19)	0.07
Hypertension (%)	0.023	(−0.01, 0.05)	0.06	0.178	(−0.09, 0.44)	0.19
Disease duration (Years)	0.170	(0.05, 0.29)	**0.01**	0.029	(−0.14, 0.20)	0.77
HbA1c (%)	1.588	(0.23, 2.94)	**0.02**	1.485	(0.89, 2.08)	**0.001**
LDL (mg/dl)	0.014	(−0.03, 0.06)	0.08	0.134	(−0.01, 0.28)	0.06
Cholesterol (mg/dl)	1.325	(0.45, 2.20)	**0.03**	1.857	(0.91, 2.80)	**0.0004**
HDL (mg/dl)	0.012	(−0.33, 0.35)	0.96	0.231	(−0.35, 0.81)	0.42
Serum C reactive protein (mg/L)	0.018	(−0.07, 0.11)	0.76	0.832	(−0.63, 2.30)	0.67
FBG (mmol/L)	0.075	(0.01, 0.14)	**0.01**	0.631	(0.22, 1.04)	**0.04**
BUN (mmol/L)	0.061	(−0.14, 0.26)	0.53	0.045	(−0.20, 0.29)	0.34
Serum creatinine(mg/dL)	0.942	(−0.11, 1.99)	0.08	1.321	(0.02, 2.62)	0.05
UA (mmol/L)	0.045	(−0.19, 0.28)	**0.06**	0.962	(−0.35, 2.28)	0.15
RBG (mmol/L)	1.607	(0.30, 2.91)	**0.01**	1.891	(1.08, 2.70)	**0.001**

**Notes.**

**R^2^ (Serum Zinc Model)** = 0.43

**R^2^ (Serum Chromium Model) =** 0.48

**Collinearity Diagnostics** All variables had VIF < 2.5, indicating no multicollinearity.

**Assumption Checks** Residuals were approximately normally distributed. Homoscedasticity and linearity assumptions were met based on residual and Q-Q plots.

Bold Styling values that the results are significant (0.05).

## Discussion

Despite the fact that trace elements are only present in small quantities in the human body, their significance to health cannot be overstated. Prior studies have highlighted the essential role of trace elements in metabolic regulation, particularly in the context of diabetes mellitus (DM). Trace element homeostasis is known to contribute to glycemic control and reduce oxidative and inflammatory damage (Kumaravelu, Shanmugam & Jayakodi, 2025; Siddiqui, Bawazeer & Scaria Joy, 2014). Among these, zinc and chromium have been widely studied due to their involvement in insulin action and glucose metabolism. Zinc functions as cofactor for many enzymes, including those insulin synthesis, storage, and secretion, while also influencing cellular processes such as apoptosis, differentiation, and signaling (Kloubert & Rink, 2015). Chromium, on the other hand, enhances insulin sensitivity and plays a role in lipid and carbohydrate metabolism, making it particularly relevant in obese or insulin-resistant individuals (Zhao et al., 2022). Our findings reinforce the importance of zinc and chromium in the pathogenesis of T2DM complications. Lower serum zinc and chromium levels were consistently observed in T2DM patients compared to healthy controls, with the most pronounced reductions occurring in patients with diabetic neuropathy and CVD, respectively. While these findings are consistent with previous reports (Al-Janabi et al., 2023; Luo et al., 2015), our study adds nuance by linking these deficiencies to specific complications. For instance, logistic regression identified serum zinc as an independent predictor of T2DM complications, supporting the potential role of trace elements as biomarkers of disease progression.

Notably, zinc deficiency was most evident in patients with diabetic neuropathy (*p* = 0.0004), while chromium was significantly reduced in those with CVD (*p* = 0.0001). These associations suggest a possible mechanistic link that zinc may contribute to neuroprotective and anti-inflammatory processes in peripheral nerves, while chromium’s impact on lipid metabolism and endothelial function may influence cardiovascular risk. However, these interpretations remain speculative without direct mechanistic data. Clinical correlations must be approached with caution, particularly given the cross-sectional design of this study. The observed association between lower zinc levels and higher HbA1c aligns with previous findings (Gouaref et al., 2024; Xu, Wen & Ye, 2024), suggesting a possible role in oxidative stress modulation and glycemic regulation. Chromium deficiency also correlated with elevated HbA1c and dyslipidemia, particularly in patients with diabetic CVD. While experimental studies suggest that chromium supplementation may improve glucose metabolism and lipid profiles (Farrokhian et al., 2020; Feng et al., 2021), the clinical benefit remains unproven in large-scale interventional studies. Thus, claims regarding supplementation must be interpreted as hypothesis-generating rather than conclusive. Clinical profiles varied by complication subgroup. Diabetic CVD patients exhibited altered lipid profiles and elevated C-reactive protein levels, which may reflect underlying inflammation and oxidative stress (Boye et al., 2023; Nanayakkara et al., 2018). Interestingly, HDL-C levels were unexpectedly higher in the diabetic group compared to controls. This paradox may reflect statin use, dietary changes, or lifestyle interventions among some patients, as previously documented (Soroush et al., 2024). Future studies should collect detailed medication and dietary histories to contextualize these findings.

Diabetic nephropathy patients demonstrated significant alterations in renal markers (urea, creatinine, and uric acid), consistent with impaired kidney function. This is particularly relevant given that renal dysfunction can confound trace element measurements due to changes in excretion and redistribution. Therefore, the association between trace element status and diabetic nephropathy should be interpreted cautiously. Similarly, diabetic neuropathy patients showed higher rates of hypertension and poor glycemic control (elevated HbA1c, lower HDL), reinforcing the complex interplay of metabolic and vascular dysfunction in neuropathy. Correlation and multivariate regression analyses revealed significant associations between trace element levels and clinical risk factors. Serum zinc was positively associated with disease duration, HbA1c, FBG, total cholesterol, and RBG, while chromium correlated with HbA1c, creatinine, and similar metabolic markers. Importantly, a positive zinc–chromium correlation was observed, supporting the hypothesis that their absorption and metabolism may be interrelated. Despite overall deficiency, some patients with worse glycemic control exhibited relatively higher trace element levels. This may reflect compensatory mechanisms such as metallothionein upregulation, altered absorption, or redistribution from tissue stores (Franco & Canzoniero, 2024; Stanojević et al., 2025; Sun et al., 2023). These complex patterns highlight the need for deeper mechanistic studies on trace element metabolism in T2DM.

The novelty of our study lies in its comprehensive assessment of zinc and chromium status across clinically distinct T2DM complication groups within a Pakistani population. Few studies have explored this degree of stratification, particularly in the South Asian context where dietary patterns, micronutrient intake, and genetic predispositions differ significantly from Western populations. However, several limitations should be noted. First, the cross-sectional nature of this study prevents causal inference. Second, the relatively small sample size limits statistical power, particularly in subgroup comparisons. Given the cross-sectional design of this study, causal inferences cannot be drawn. Accordingly, the observed associations between serum trace element deficiencies and specific diabetic complications should be interpreted with caution. These findings warrant further validation through longitudinal cohort studies or randomized controlled trials to explore potential causality and therapeutic implications. Third, as a retrospective analysis, the study lacked information on dietary intake, zinc and chromium intake, medication use (such as statins, antihypertensive, and metformin), and renal function markers in non-nephropathy groups, all of which could influence trace element status and potentially confound the observed associations. Future studies should incorporate dietary and supplement data to clarify these relationships. Moreover, renal impairment in nephropathy may confound trace element interpretation, as altered excretion and protein binding can skew serum measurements.

## Conclusions

This study highlights a significant association between reduced serum levels of zinc and chromium and the severity of T2DM, particularly in patients with neuropathy and cardiovascular complications. These findings support the potential involvement of trace element imbalances in the pathophysiology of T2DM. However, given the observational design, causal inferences cannot be made. While monitoring these elements may offer clinical insight into disease progression, recommendations for supplementation should be approached cautiously. Further longitudinal and interventional studies are necessary to clarify their mechanistic roles and therapeutic potential in T2DM management.

## Supplemental Information

10.7717/peerj.20184/supp-1 Supplemental Information 1 Raw data

10.7717/peerj.20184/supp-2 Supplemental Information 2 Raw data with all participants

10.7717/peerj.20184/supp-3 Supplemental Information 3 Questionnaire, Informed Consent, and Data

10.7717/peerj.20184/supp-4 Supplemental Information 4 Appendix

10.7717/peerj.20184/supp-5 Supplemental Information 5 STROBE Checklist

## Additional Information and Declarations

### Data Availability

The following information was supplied regarding data availability: The data is available in the Supplemental Files.
