# Supplementary material for: Clinical correlation of serum zinc and chromium levels in patients with type 2 diabetes mellitus and complications in Pakistan: a retrospective study
Source: PeerJ. 2026 Jan 28;14:e20184. doi: 10.7717/peerj.20184 (PMC12860277; doi:10.7717/peerj.20184)
Supplement: Supplemental Information 3 [file peerj-14-20184-s003.docx]

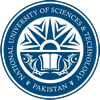


**National University of Sciences and Technology (NUST)**

### Atta-ur-Rahman School of Applied Biosciences (ASAB)

**Performa for Clinical Investigation of Trace elements levels in blood serum associated with Type 2 diabetes mellitus Incidence and Complications.**

Patient’s Name: Specific to person Age: _ Specific to person ___

Sample ID: For labeling Date: ____________

Gender (M /F): _____________________ Contact No: _______ __ ______

Occupation: **___________________** Income ____ ________ __

Address: ___________ _______________________________________ _ _

______________ ______________________________________

**CLINICAL PROFILE**

|  | YES | NO |
| --- | --- | --- |
| Family History of Type 2 Diabetes |  |  |
| Family History of Cardiovascular diseases |  |  |
| Family History of Ocular diseases |  |  |
| Family History of Renal diseases |  |  |
| Family History of Neurological diseases |  |  |

Smoking: Yes _____ No _ _ __ Quitted ________

Height: _variable_________ Weight: _________________

Age at diagnosis _______years________

Duration of Disease 1) < 5 Yrs 2) 5–10 Yrs 3) >10 Yrs

Type of treatment for T2D__Insulin dependent /Non-insulin dependent

| HbA_1_C level | Mentioned in results |
| --- | --- |
| Fasting plasma glucose level | Mentioned in results |
| Random blood glucose level | Mentioned in results |
| Albuminuria / Serum Albumin | Mentioned in results |
| Serum Urea | Mentioned in results |
| Serum Creatinine | Mentioned in results |
| Serum ALP | Mentioned in results |
| Serum ALT | Mentioned in results |
| Serum C-reactive Protein levels | Mentioned in results |
| Serum Cholesterol level | Mentioned in results |
| Serum HDL level | Mentioned in results |
| Serum LDL level | Mentioned in results |
| Serum Triglyceride levels | Mentioned in results |

**History of general health/ associated defects (congenital/ acquired):**  ____ ___ __ _

**Diabetes associated defects: __**  **_________**

**Note:** Consent of patient/guardian has been taken in his /her own language and all the information will be confidential.

______________________

Signature

Physician / Medical doctor

_______________________

Countersigned by Principal Investigator:

(Dr. Attya Bhatti)

ASAB, NUST

**Table 1 for comparing anthropometric and biochemical parameters between all complications**

| Clinical Parameters | Without any complication  (n=20) | With diabetic retinopathy  (n=24) | With diabetic neuropathy  (n=13) | With diabetic nephropathy  (n=11) | With diabetic CVD  (n=25) | Control | *P value* |
| --- | --- | --- | --- | --- | --- | --- | --- |
| Males (%) | 08 (40%) | 12 (50%) | 07 (53.8%) | 06 (54.5%) | 14 (56%) | 22  (53.4%) | 0.0986 |
| Females (%) | 12 (60%) | 12 (50%) | 06 (46.1%) | 05 (45.4%) | 11 (44%) | 21  (46.5%) | 0.7537 |
| Age (Years) | 56.7 ±10.19 | 60.3 ±13.3 | 52.6 ±11.8 | 55.9 ±12.9 | 62.6±11.4 | 53.4±9.1 | 0.9876 |
| BMI | 22.9± 6.9 | 23.1±7.3 | 20.81±5.5 | 22.7±6.1 | 25.9±12.2 | 21.5±4.5 | 0.0678 |
| Disease duration (Years) | 5.55 ±4.58 | 9.16±7.16 | 8 ±5.55 | 7.18±5.25 | 10.1±7.33 | --------- | -------- |
| FBG (mg/dl) | 121±42.6 | 153±56.2 | 164±64.6 | 145±50.9 | 138±46.9 | 90±39.3 | 0.8905 |
| RBG (mg/dl) | 275±122 | 241±147 | 276±178 | 230±53.3 | 243±111 | 6.7(1.1-1.4) | 0.0890 |
| HbA1c (%) | 8.6±2.54 | 9.44±1.91 | 9.8±2.02 | 8.9±1.85 | 9.11±1.61 | 5.2±0.5 | 0.0578 |
| Smoking n (%) | 4(20%) | 6(30%) | 8(40%) | 5(25%) | 4(20%) | 3(15%) | 0.4534 |
| Hypertension (%) | 6(30%) | 8(40%) | 10(50%) | 4(20%) | 6(30%) | 5(25%) | 0.0894 |
| BUN (mmol/L) | 31±7.9 | 35±11.8 | 38±9 | 41±8 | 33±14.1 | 29±5 | 0.045 |
| Serum creatinine (mmol/L) | 1.2±0.8 | 1.6±1.2 | 1.1±0.7 | 1.9±1.4 | 1.5±1.6 | 0.9±0.2 | 0.023 |
| Serum C reactive protein | 2.5±2.2 | 2.9±2.3 | 2.8±2.3 | 2.8±2.6 | 3.1±2.7 | 2.5±2.0 | 0.065 |
| Cholesterol (mg/dl) | 190 | 200 | 219 | 210 | 245 | 158 | 0.044 |
| HDL (mg/dl) | 49 | 52 | 53 | 49 | 46 | 39 | 0.042 |
| LDL (mg/dl) | 150 | 155 | 153 | 158 | 162 | 152 | 0.8357 |
| Triglycerides (mg/dl) | 202 | 214 | 223 | 201 | 238 | 149 | 0.673 |
| UA (mmol/L) | 0.53 | 0.56 | 0.45 | 0.67 | 0.49 | 0.25 | 0.051 |

**Anthropometric and biochemical parameters comparison between all complications**

**Table 2: Correlation between serum zinc and chromium level in diabetic CVD patients.**

| Item | Serum zinc | | Serum chromium | |
| --- | --- | --- | --- | --- |
|  | **r** | ***P*** | **r** | ***P*** |
| Age (Years) | -0.00389 | 0.07 | 0.02691 | 0.04 |
| BMI (kg/m^2^) | 0.00031 | 0.09 | 0.00460 | 0.07 |
| Hypertension (%) | -0.00745 | 0.05 | -0.03239 | 0.03 |
| Smoking | 0.00534 | 0.05 | 0.00344 | 0.02 |
| HbA1c (%) | -0.00185 | 0.01 | -0.02246 | 0.004 |
| LDL (mg/dl) | -0.00014 | 0.001 | 0.00134 | 0.011 |
| Cholesterol (mg/dl) | 0.00234 | 0.04 | -0.02373 | 0.0001 |
| HDL (mg/dl) | 0.04782 | 0.43 | -0.05321 | 0.51 |
| Serum C reactive protein | 0.02437 | 0.24 | 0.00342 | 0.005 |

**Table 2: Correlation between serum zinc and chromium level in diabetic CVD patients.** Zinc and chromium level were correlated with different demographical and clinical parameters. BMI, Body mass index; HbA1c, Glycated haemoglobin; LDL, Low density lipoprotein.

**Table 3: Co-relation between serum zinc and chromium level in T2DM patients with retinopathy**

| Item | Serum zinc | | Serum chromium | |
| --- | --- | --- | --- | --- |
|  | **r** | ***P*** | **r** | ***P*** |
| Age (Years) | 0.01131 | 0.01 | 0.00045 | 0.09 |
| BMI (kg/m^2^) | 0.11534 | 0.95 | -0.01563 | 0.05 |
| Hypertension (%) | -0.00345 | 0.03 | -0.06741 | 0.06 |
| Smoking | 0.00442 | 0.98 | 0.03488 | 0.08 |
| HbA1c (%) | -0.00121 | 0.04 | -0.07431 | 0.04 |
| LDL (mg/dl) | -0.00067 | 0.002 | 0.00934 | 0.06 |
| Cholesterol (mg/dl) | 0.00349 | 0.05 | -0.03245 | 0.02 |
| HDL (mg/dl) | 0.0674 | 0.568 | -0.0943 | 0.031 |
| Serum creatinine | -0.05643 | 0.0977 | 0.004521 | 0.063 |

**Table 3 : Co-relation between serum zinc and chromium level in T2DM patients with retinopathy** Zinc and chromium level were correlated with different demographical and clinical parameters. BMI, Body mass index; HbA1c, Glycated haemoglobin; LDL, Low density lipoprotein.

**Table 4: Co-relation between serum zinc and chromium level in diabetic neuropathy patients**

| Item | Serum zinc | | Serum chromium | |
| --- | --- | --- | --- | --- |
|  | **r** | ***P*** | **r** | ***P*** |
| Age (Years) | 0.00632 | 0.045 | 0.00245 | 0.063 |
| BMI (kg/m^2^) | 0.01544 | 0.043 | -0.04321 | 0.543 |
| Hypertension (%) | -0.00045 | 0.001 | -0.08432 | 0.065 |
| FBG (mmol/ | 0.02346 | 0.053 | 0.04534 | 0.983 |
| HbA1c (%) | -0.01134 | 0.053 | -0.06796 | 0.089 |
| LDL (mg/dl) | -0.00067 | 0.003 | 0.00934 | 0.065 |
| BUN (mmol/L) | 0.00349 | 0.076 | 0.03245 | 0.590 |
| HDL (mg/dl) | 0.06743 | 0.568 | -0.09434 | 0.031 |
| Serum creatinine | 0.05343 | 0.457 | 0.07897 | 0.089 |

**Table 4: Co-relation between serum zinc and chromium level in diabetic neuropathy patients**. Zinc and chromium level were correlated with different demographical and clinical parameters. BMI, Body mass index; HbA1c, Glycated haemoglobin; LDL, Low density lipoprotein,FBG; fasting blood glucose;HDL,High density lipoprotein.

**Table 5 : Co-relation between serum zinc and chromium level in diabetic nephropathy patients.**

| Item | Serum zinc | | Serum chromium | |
| --- | --- | --- | --- | --- |
|  | **r** | ***P*** | **r** | ***P*** |
| Age (Years) | 0.00178 | 0.019 | 0.03548 | 0.063 |
| BMI (kg/m^2^) | 0.07800 | 0.654 | -0.08946 | 0.543 |
| Hypertension (%) | -0.00931 | 0.001 | 0.09429 | 0.582 |
| FBG (mmol/L) | 0.04527 | 0.068 | 0.08325 | 0.839 |
| HbA1c (%) | -0.09013 | 0.05 | -0.09092 | 0.040 |
| UA (mmol/L) | -0.00797 | 0.005 | 0.08930 | 0.068 |
| BUN (mmol/L) | 0.06749 | 0.054 | 0.04545 | 0.059 |
| RBG (mmol/L) | -0.06749 | 0.136 | -0.04l34 | 0.759 |
| Serum creatinine | -0.08143 | 0.025 | 0.05428 | 0.083 |

**Table 5: Co-relation between serum zinc and chromium level in diabetic nephropathy patients.** Zinc and chromium level were correlated with different demographical and clinical parameters. BMI, Body mass index; HbA1c, Glycated haemoglobin; FBG, Fasting blood glucose,RBG; Random blood glucose;UA,Uric acid;BUN,blood urea nitrogen**.**

INFORMED Consent Form

**Trace elements profiling in blood serum of Type 2 Diabetes Mellitus**

**AIMS AND OBJECTIVES**

To elucidate the role of Trace elements imbalances in Type 2 Diabetes mellitus pathogenesis and its associated complications

**SAMPLE COLLECTION**

For participants with Type 2 diabetes mellitus and associated complications, a 5-10 ml blood sample will be collected. This includes individuals diagnosed with type 2 diabetes mellitus, those experiencing complications such as joint deformities, inflammation, and systemic manifestations. Additionally, participants with renal diseases, cardiovascular complications related to type 2 diabetes mellitus.

Informed Consent

You are being asked to participate in a research study to study genetic basis of Type II Diabetes and its associated complications. You will be asked to donate 5-10ml of blood. This will not cause any physical injury. Your samples will be preserved in the laboratory and will be tested for genetic variability of specific genes involved in Type II Diabetes. Your identity in this study will be protected. You can withdraw your participation at any time in the course of this study. This research project will be carried out solely on a non-commercial basis. Your participation is voluntarily. The scientific information will only be shared among the collaborating scientists. The results of the study if novel or of medical interest will be published in scientific journals without disclosing your identity.

اجازت نامہ

مجوزہ تحقيقي منصوبہ آپ کے اندر وقوع پزیر ہونے والی جینیاتی بہروپتا کی وجہ سے وقوع ہونے والی ذیابیطس قسم دوم میں تبدیلی معلوم کرنے کیلیے خالصتاََ غیر تجارتی بنیادوں پر ترتیب دیا گیا ہے۔ آپکو۱۰۔۵ ملی لیٹر خون عطیہ کرنے کی درخواست کی جاتی ہے۔ خون دینے سے آپکو کِسی قِسم کازخم یا درد نہیں ہو گا۔ آپ کي شناخت کو مکمل طور پر صيغۂ راز ميں رکھا جاۓ گا. آپ کا ديا ہوا عطيہ تجربہ گاہ ميں محفوظ رکھا جاۓ گا اور ہم ان خاص جينياتي تبديليوں کا مشاہدہ کرتے ہوۓ ذیابیطس قسم دوم کو سمجھنے کی کوشش کریں گے۔ آپ اس تحقيق کے دوران کسي بھي وقت اپني شموليت سے دستبردار ھو سکتے ہيں. آپ کي شموليت رضاکارانہ ہے. دوران تحقيق اگر ضروري ہوا تو آپکی ذیابیطس قسم دوم سے متعلق مشاورت بھی کی جاے گی۔ حاصل شدہ ساءنسي معلومات کا تبادلہ صرف تحقيق ميں شامل ساءنسدانوں کے مابين کيا جاۓ گا. تحقیق سے حاصل ہونےوالے انوکھے نتا یجٔ کو سائنسی جریدے میں آپکی شناخت کو پوشیدہ رکھتے ہوے شاٰئع کیا جائے گا۔

**I hereby confirm that I fully understand what has been stated above. I voluntarily donate blood sample**

**from myself / and from my family for research purposes only.**

ميں تصديق کرتا یا کرتی ھوں کہ جو کچھ بھي مجھ سے بيان کيا گيا ھے، ميں اسے مکمل طورپر سمجھ گیا یا گیٔی ھوں. نيز ميں اپنے خون کا نمونہ رضاکارانہ طور پر صرف تحقيق کے لۓ بطور عطيہ ديتا/ديتي ھوں مجھے ميرے تمام سوالات کے جواب مل گۓ ہيں اور في الوقت ميرے ذہن ميں کوٸ اور سوالات نہيں.

**Signature/Thumb impression of the participant/head of the family: _________________________**

**Name:** _____________________________ **Participant ID**____________________ **Contact no.______________________**
