# Supplementary material for: Clinical correlation of serum zinc and chromium levels in patients with type 2 diabetes mellitus and complications in Pakistan: a retrospective study
Source: PeerJ. 2026 Jan 28;14:e20184. doi: 10.7717/peerj.20184 (PMC12860277; doi:10.7717/peerj.20184)
Supplement: Supplemental Information 4 [file peerj-14-20184-s004.docx]

**Appendix**

**HbA1c (Glycated Hemoglobin):** Reflects average blood glucose over the past 2–3 months. Used to assess long-term glycemic control

**FBG (Fasting Blood Glucose):** Blood sugar level measured after fasting for at least 8 hours.

**RBG (Random Blood Glucose):** Blood sugar level measured at any time, regardless of meals.

**Serum Creatinine:** Marker of kidney function; elevated levels may indicate renal impairment.

**Total Cholesterol:** Sum of all cholesterol types in the blood; high levels increase cardiovascular risk.

**HDL (High-Density Lipoprotein):** "Good" cholesterol that helps remove excess cholesterol from blood vessels.

**LDL (Low-Density Lipoprotein):** "Bad" cholesterol that can build up in arteries and raise heart disease risk.

**Triglycerides:** Blood fats that increase with insulin resistance and contribute to cardiovascular risk.

**Uric Acid:** Metabolic waste product; elevated levels may indicate kidney dysfunction or metabolic stress.

**Urea:** Waste product of protein metabolism; used to evaluate kidney function.

**Serum C - reactive protein (CRP):** Inflammatory marker; high levels may reflect systemic inflammation linked to metabolic disorders.
